# How Far Are Non-Viral Vectors to Come of Age and Reach Clinical Translation in Gene Therapy?

**DOI:** 10.3390/ijms22147545

**Published:** 2021-07-14

**Authors:** Myriam Sainz-Ramos, Idoia Gallego, Ilia Villate-Beitia, Jon Zarate, Iván Maldonado, Gustavo Puras, Jose Luis Pedraz

**Affiliations:** 1 NanoBioCel Group, Faculty of Pharmacy, University of the Basque Country UPV/EHU, 01006 Vitoria-Gasteiz, Spain; miriam.sainz@ehu.eus (M.S.-R.); idogia.gallego@ehu.eus (I.G.); aneilia.villate@ehu.eus (I.V.-B.); jon.zarate@ehu.eus (J.Z.); imaldonado002@ikasle.ehu.eus (I.M.); 2 Networking Research Centre of Bioengineering, Biomaterials and Nanomedicine (CIBER-BBN), 28029 Madrid, Spain; 3 Bioaraba, NanoBioCel Resarch Group, 01009 Vitoria-Gasteiz, Spain

**Keywords:** non-viral vectors, gene therapy, nanotechnology, gene delivery, clinical translation

## Abstract

Efficient delivery of genetic material into cells is a critical process to translate gene therapy into clinical practice. In this sense, the increased knowledge acquired during past years in the molecular biology and nanotechnology fields has contributed to the development of different kinds of non-viral vector systems as a promising alternative to virus-based gene delivery counterparts. Consequently, the development of non-viral vectors has gained attention, and nowadays, gene delivery mediated by these systems is considered as the cornerstone of modern gene therapy due to relevant advantages such as low toxicity, poor immunogenicity and high packing capacity. However, despite these relevant advantages, non-viral vectors have been poorly translated into clinical success. This review addresses some critical issues that need to be considered for clinical practice application of non-viral vectors in mainstream medicine, such as efficiency, biocompatibility, long-lasting effect, route of administration, design of experimental condition or commercialization process. In addition, potential strategies for overcoming main hurdles are also addressed. Overall, this review aims to raise awareness among the scientific community and help researchers gain knowledge in the design of safe and efficient non-viral gene delivery systems for clinical applications to progress in the gene therapy field.

## 1. Introduction

**Figure 1:**
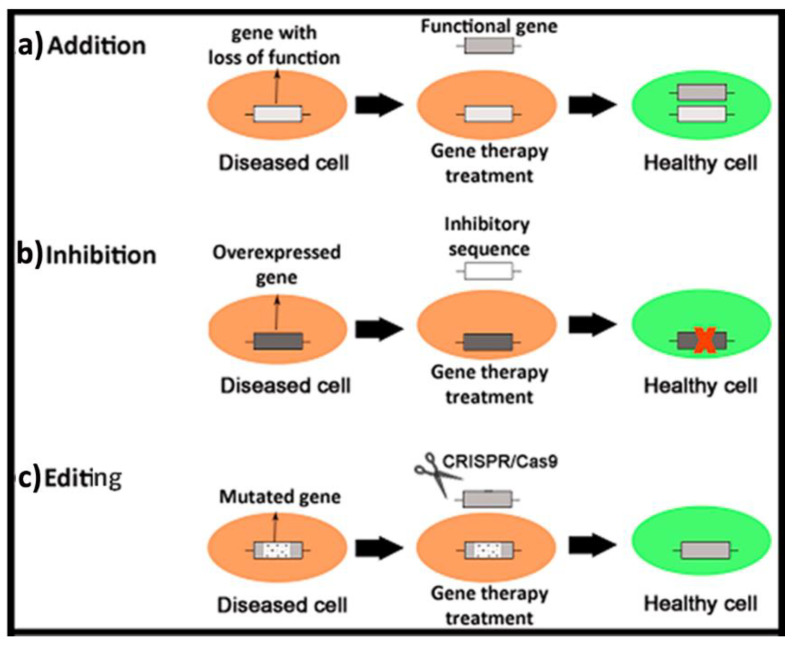
Brief schematic representation of three different genetic material-based approaches to face human diseases. (**a**) Gene addition therapy. (**b**) Gene inhibition therapy. (**c**) Genome editing.

The main concept of gene therapy is quite simple and overall relies on the delivery of exogenous genetic material into target cells to modulate the expression of an altered genome. Basically, three different approaches can be identified (Figure 1). In the case of *gene addition therapy*, a “healthy” copy of the gene is administered to recover the functionality of the affected cells. This strategy can be suitable to face diseases caused by mutations with loss of function [1]. For instance, the autosomal recessive cystic fibrosis disease caused by a deletion of the phenylalanine at the position 508 of the CFTR (cystic fibrosis transmembrane conductance regulator) protein [2]. However, in the case of a mutation that overexpresses genes, the aim is to administer an inhibitory sequence to knock out the expression of the mutated gene [3]. This strategy is referred to as *gene inhibition therapy* and can be applied, for instance, to face autosomal dominant retinitis pigmentosa secondary to specific mutations in the pre-mRNA splicing-factor gene PRPF31 [4]. The third approach, named as *genome editing*, incorporates specific genome editing tools to repair mutations in the genome with gain or loss of function [5]. This strategy has been successfully used in combination with iPSC technologies to combat human β-thalassemia disease in mice [6].

The main characteristics and composition of the genetic cargo strongly depend on the gene therapy approach used (Table 1). For instance, in the case of the *gene addition* approach, which is classically used to face genetic disorders that follow an autosomal recessive inheritance pattern, the most common polynucleotide used is referred to as plasmid (pDNA). Such a plasmid is a circular and double-stranded DNA construct, typically between 1.5 and 20 kbs, that drives the transient transgene expression in the nucleus of target cells, encoding the protein of interest [7]. Typically, a transfection mediated by conventional pDNAs is moderated and only active during 1–2 months. However, smaller versions of a conventional pDNA, known as minicircle DNA (mcDNA, 2–6 kbs) or micro-intronic plasmid (2–4 kbs), can improve transgene expression by 10- to 100-fold and prolong the effect for some years [8,9]. Another commonly used polynucleotide in the *gene addition* approach is the single strand messenger RNA (mRNA). Nowadays, this strategy holds great promise, especially in the vaccine research area, since two vaccines produced by Moderna and Pfizer/BioNTech companies have been recently approved by the European Medicines Agency (EMA) to fight against the severe acute respiratory syndrome coronavirus 2 (SARS-CoV-2) infection and the resulting coronavirus disease (COVID-19). As in the case of pDNA, the effect mediated by mRNAs is also transient and the stability in plasma is even lower, around 1 h. The less tight conformation of RNA, which allows an easier access of the degradative enzymes, and also the presence of hydroxyl groups in the main structure, which enhances hydrolyzation of RNAs [10,11], significantly contribute to decrease the stability. However, the main advantages of RNA-based gene therapy compared to pDNA include a safer profile, since it decreases the risk of mutagenesis and immunogenicity and a more efficient modulation of target gene expression because the place of action of this genetic cargo is in the cytoplasm [12]. Therefore, there is no need to get access into the nucleus of cells, which is classically considered as one of the main bottlenecks of plasmid-based expression systems [13]. 

In contrast to *gene addition strategy*, the *inhibition approach* can be used when the main goal is to silence the expression of an altered gene that has a gain of function mutation. This scenario is common in genetic disorders that follow an autosomal dominant inheritance pattern [14]. In this case, the inhibitory sequence can also have a single strand RNA-based structure, such as the microRNA (miRNA), or a double strand RNA structure, such as the small interfering RNA (siRNA). Typically, these structures inhibit the translation of mRNA in the RNA-induced silencing complex (RISC) of the cytoplasm in a transitory way to avoid the expression of the target gene [15]. Interestingly, other synthetic and smaller single strand RNA/DNA structures, known as antisense oligonucleotides (AON), can also inhibit mRNA translation by a different mechanism of action. In this case, the oligonucleotide sequence interferes with pre- and mRNA in the nucleus or cytoplasm through a complementary hybridization mechanism that enhances specificity but with lower knockdown efficiency [16]. 

Apart from *gene addition* and *gene inhibition* strategies, another approach consists of the permanent correction of the mutated gene with the use of specific genome editing tools such as those developed by the game changer CRISPR/Cas9 technology [17]. In this case, once the mutated gene is identified, different RNA guides (gRNA), which recognize 20 nucleotides of the mutated allele, can be designed and synthetized to be delivered alongside the Cas9 protein, which will cut the genome 3 nucleotides to the left side in the 5′-3′ direction of the protospacer adjacent motif (PAM) region. In this scenario, a single strand DNA sequence with the corrected mutation, typically 100 nucleotides long, can be supplied as a donor template to be incorporated in the cell genome by the homologous recombination mechanism [18]. Such CRISPR/Cas9 editing tools can be delivered in different genetic constructors such as pDNA, mRNA or ribonucleoprotein (RNP) complex, acting in different cell places [19]. 

**Figure 2:**
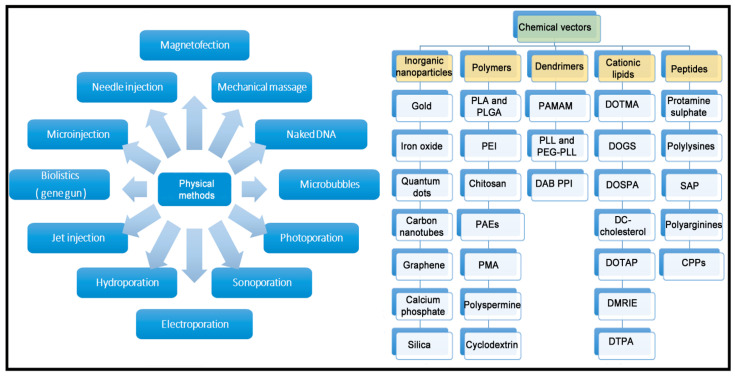
Overview of main physical and chemical methods of non-viral vectors.

Unfortunately, all the previously described genetic cargoes need to overcome both extracellular and intracellular barriers to reach the place of action. In the case of in vitro conditions, which is the simplest scenario, only intracellular barriers need to be considered. However, in the case of in vivo experimentation, the delivery process to the place of action can also be affected by additional extracellular barriers, which strongly depends on the route of administration and the organ to be treated [20]. To overcome such biological barriers, gene delivery systems are necessary, since genetic cargo by itself, in most of the cases, is not effective. Classically, gene delivery systems are divided into viral and non-viral vectors. Viral vectors are recognized by their high gene delivery efficiency. In fact, viruses have evolved along many years to infect efficiently different kinds of cells with their genetic cargo, and currently, such viruses can be easily modified in the laboratory to deliver the genetic cargo of interest into target cells, reducing their pathogenic effect [21]. As a result, most of the clinical trials, and the great majority of gene therapy drugs approved for human use by regulatory agencies, are based on viral vectors. Some examples of marketed gene therapy products that use viral vectors include Luxturna, Zolgensma, Oncorine and Imlygic, to name just a few [22,23]. However, relevant concern still remains in the research community related, over all, to their potential immunogenicity and oncogenic capacity [24,25]. In addition, previously mentioned approved drugs are highly expensive, mainly due to the intrinsic characteristics of biologic drugs [26]. Therefore, interest in non-viral gene delivery systems has recently gained momentum. A brief schematic representation of both physical and chemical methods for non-viral gene delivery is summarized in Figure 2.

**Figure 3:**
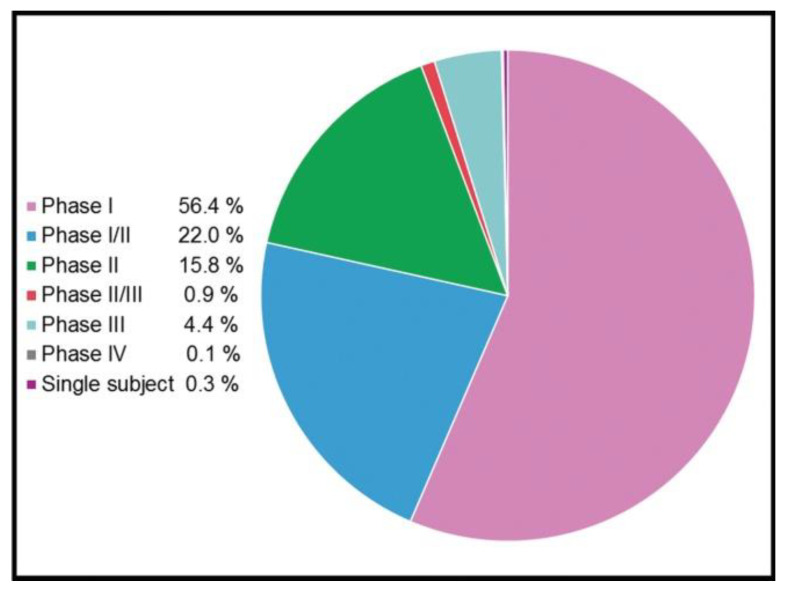
Clinical phases of 3180 ongoing trials based on gene therapy (adapted from http://www.genetherapynet.com/clinical-trials.html; accessed 1 June 2021; gene therapy clinical trial database).

Compared to their viral counterparts, non-viral vectors show some appealing properties such as lower immunogenicity, safer profile and higher genetic cargo packing capacity. In addition, non-viral vectors are cheaper and easier to manufacture and scale up [27]. Due to these obvious advantages, the gene delivery mediated by non-viral vectors is nowadays considered the cornerstone of modern gene therapy, especially for CRISPR/Cas9 delivery, where non-viral vectors predominate over viral vectors at the preclinical level [28]. In any case, although with few exceptions, this strategy has been poorly translated into clinical success. However, some promising clinical trials based on gene therapy treatments, summarized in Figure 3, are ongoing.

**Figure 4:**
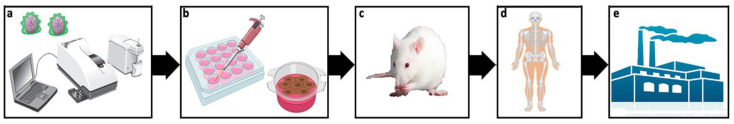
Journey of non-viral vectors from lab to bench. Initially potential non-viral vectors are physicochemically characterized (**a**) before performing both in vitro (**b**) an in vivo (**c**) biological studies. Most promising formulations are further evaluated in clinical trials (**d**). In case of success, the manufacturing process for commercialization starts (**e**).

In this review, some critical issues in the way to clinic application of non-viral vectors (Figure 4) and potential strategies to overcome such hurdles have been addressed. More specifically, special attention has been paid to the gene delivery efficiency and biocompatibility of non-viral vectors. Additionally, the duration of the transgene expression, along with the route of administration, the design of experimental conditions and some concerns related to the commercialization process, has also been discussed.

## 2. Gene Delivery Efficiency

A critical issue that hampers the regular application of non-viral vectors into regular medical practice is their low gene delivery efficiency [29]. In this sense, viral systems clearly surpass non-viral counterparts, probably due to their continuous evolution along millions of years, which has allowed them to get better access into the genome of the target cell, overcoming both extracellular and intracellular barriers [30]. Nowadays, most of the commercially available gene therapy-oriented drugs use recombinant viruses modified in the laboratory, such as retroviruses, lentiviruses, adenoviruses or adeno-associated viruses to shuttle their genetic cargo. However, their overall safety concerns related to the biological origin and the low genetic cargo packing capacity, along with the difficulties associated to scaling up their production and high cost of development, have contributed to exploring different gene delivery approaches based on the design of novel non-viral vectors [31]. Research on this area has quickly captured the attention of the scientific community, and, currently, this strategy represents a safer and more affordable alternative, although the gene delivery efficiency of these systems needs to be improved to reach a regular clinical practice. 

Gene delivery efficiency of non-viral vectors is typically evaluated at a preclinical level once such systems have shown appropriate physicochemical and biophysical properties, for instance, in terms of particle size, superficial charge, polydispersity, morphology and capacity to bind and protect genetic material to release it without suffering any degradation, since all these parameters can affect the transfection process [32]. Initially, and as a proof of concept, gene delivery capacity is evaluated in culture cells and, for that purpose, the expression of different reporter plasmids that encode fluorescent proteins [33] or enzymes, such as luciferase [34] or galactosidase [35], is employed to be quantitatively evaluated by different techniques. In this sense, it is worth mentioning that each reporter plasmid and corresponding assays have different sensitivity and their own metrics [36]. For example, the reporter plasmid that encodes green fluorescent protein is a good descriptor of the transfection efficiency at a single cell’s level, since results are typically expressed as the percentage of live cells that show green signal by flow cytometry [37]. However, luciferase expression provides information related to the plasmid expression in a whole population of cells, since the luminescence is normalized by the quantity of proteins in cell lysates. 

**Figure 5:**
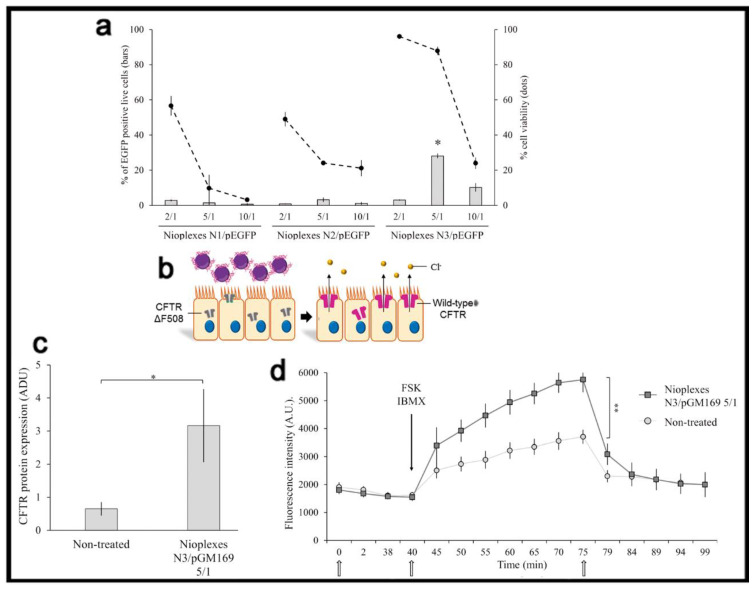
Transfection efficiency and therapeutic effect. (**a**) Percentage of live transfected CuFi-1 cells with the pEGFP reporter plasmid. (**b**) General scheme of transfection process with pGM169 therapeutic plasmid. (**c**) CFTR/β-actin protein expression determined by Western blot. (**d**) CFTR chloride channel activity determined by SPQ analysis. Reproduced with permission of [38].

When a therapeutic genetic material, instead of the reporter one, is used in in vitro conditions, it is also important to consider, from a practical point of view, the transfection efficiency value required to reach a therapeutic effect, which highly depends on the particular application and disease. For instance, in the case of cystic fibrosis, an autosomal recessive disorder caused by the dysfunction of the CFTR gene, 28% of living human cystic fibrosis airway epithelial cells (CuFi-1) were transfected with the pEGFP reporter plasmid, using a lipid-based non-viral vector, named as N3 [38]. Such formulation reported a 5-fold increase of CFTR protein expression in transfected versus non-transfected cells with the pGM169 therapeutic plasmid, which led to 1.5-fold increment of the chloride channel functionality, exceeding the value required to get a therapeutic benefit (Figure 5).

Such in vitro studies are typically used as a screening methodology to select the non-viral vector candidates that show better performance before conducting in vivo studies, in accordance with the principle of the three Rs (replacement, reduction and refinement of animal labs). This sequential approach is aimed to reduce the number of animals used in in vivo experiments. However, it should also be borne in mind that a direct correlation between in vitro and in vivo results in terms of gene delivery efficiency does not always exist, essentially because experimental conditions in each scenario are quite different [37]. As a consequence, some readjustments in terms of the composition of the formulation, preparation methods, doses or volumes to be administered need to be performed to succeed in in vivo experiments [39].

In any case, the transfection efficiency of non-viral vectors is highly related to their cytotoxic effect, which is also a highly cell-dependent process [40]. Therefore, a suitable balance between the transfection efficiency value required to obtain a therapeutic effect and the cytotoxic effect needs to be acquired for each clinical application to enhance translation of non-viral vectors to the regular medicine practice. Such a toxic effect of non-viral vectors depends on many physicochemical parameters that affect the gene delivery process such as particle size, morphology and zeta potential of complexes [41]. In addition, the elaboration method, along with the intrinsic properties of the materials used to obtain the different kinds of non-viral vectors, can impact on the final cytotoxic effect, depending, for instance, on the degradation rate or the persistence along the time in organs and tissues [42]. It should also be kept in mind that the persistence and accumulation of metabolites that come from the degradation of different compounds present in non-viral vector formulations can also induce an inflammatory process and, therefore, cause toxicity. Nevertheless, the cytotoxic effect does not only depend on the non-viral vector’s compounds. The genetic material that is aimed to be delivered can also be toxic, considering, for instance, the bacterial origin of many plasmids that can enhance the induction of undesired immune responses and the secretion of proinflammatory cytokines [43]. Interestingly, small plasmidic cassettes as mcDNA have been recently developed to mitigate some disadvantages associated with the use of conventional plasmids [31]. Such mcDNAs contain a minimal expression cassette, where the bacterial backbone DNA has been eliminated, which reduces the unwanted immunogenic responses and enhances the transfection efficiency due to the reduced size of this CpG-free genetic material [8,44]. The cytotoxic effect of the non-viral vectors can be qualitatively evaluated by different techniques based on microscopy analyses [45]. However, normally, quantitative analysis of toxicity is assessed by means of a broad spectrum of cell viability/cytotoxicity colorimetric available kits, such as, for instance, CCK8, MTT assay and Alamar Blue^TM^, or by mean of flow cytometer analysis [46,47,48]. In this sense, it is worth mentioning that many chemical compounds that are present in non-viral vector formulations can interfere with the previously described colorimetric assays, providing confusing results. In the case of flow cytometer analysis, fluorescent dyes such as ethidium homodimer-1, propidium iodide or 7-Amino-actinomycin D (7-AAD) are normally used to stain and analyze dead cells, which should be excluded from the final transfection efficiency results [49].

To reduce the cytotoxic effect of non-viral vector formulations, many natural compounds, such as cholesterol [50], lycopene [39] or squalene [33], can be incorporated into lipid vesicles as “helper” components. In addition, some non-ionic surfactants, such as polysorbate 80, can also reduce the toxic effect of cationic lipids [51]. Although the use of cationic materials, such as the mentioned cationic lipids or polycationic polymers, facilitates the complexation with the negatively charged nucleic acids for gene delivery as well as cellular internalization, an excess of positive charge can have detrimental effects on cell viability. Hence, other strategies to avoid cationic vectors, and thus cytotoxicity, have been developed for nucleic acid delivery [52]. In the case of polymeric-based non-viral vector formulations, *stimuli responsive polymers*, also knowns as *intelligent polymers*, represent an appealing approach to enhance not only biocompatibility of the formulation but also the specificity and the duration of the gene expression [53]. These particular polymers can modify their biological performance in response to small environmental changes of physicochemical parameters such as pH value, temperature or ionic strength to name just some of the most relevant ones [54].

## 3. Duration of Gene Expression

Another main reason that limits the clinical application of non-viral vectors into regular medical practice is the loss of transgene expression over time in clinical trials. A transgene expression can decrease with time because of many causes such as the inactivation of the genetic material by nucleases, the loss of activity by recombination processes, the ineffective distribution into intracellular vesicles, or even the recognition and subsequent silencing of foreign DNA by the host immune system [55]. In this sense, while the retroviral and lentiviral vectors do integrate into the host cell genome, providing a long-lasting effect [56,57], the main reason for adeno-associated viruses (AAV) vectors to provide sustained transgene expression is not integration. AAV vectors barely integrate into the genome unlike wild-type AAV. In contrast, an AAV vector genome persists in the host cell nucleus as episomal concatemers that are highly resistant to nucleases. The fact that AAV genomes are diluted over time as the cell undergoes repeated rounds of replication, with the rate of transgene loss dependent on the turnover rate of the transduced cell [58], is a proof of the occurrence. For instance, commercially available Luxturna drug delivers by means of an AAV type 2 a healthy copy of the RPE65 gene into the subretinal space of patients affected by retinitis pigmentosa and Leber congenital amaurosis. Despite the high cost of the treatment, around $850,000, only one injection is required to complete the treatment, due to the long-lasting effect obtained, in slow dividing cells of the retina. This fact is particularly relevant in the case of invasive routes of administrations, such as intravitreal, subretinal or administrations, into the cerebral cortex after craniotomy. In this scenario, repeated administrations could increase the after-care cost due to additional hospital visits [59], and, in many cases, jeopardize the acceptance of these aggressive gene delivering routes because of the cumbersome approach and related side effects. 

**Figure 6:**
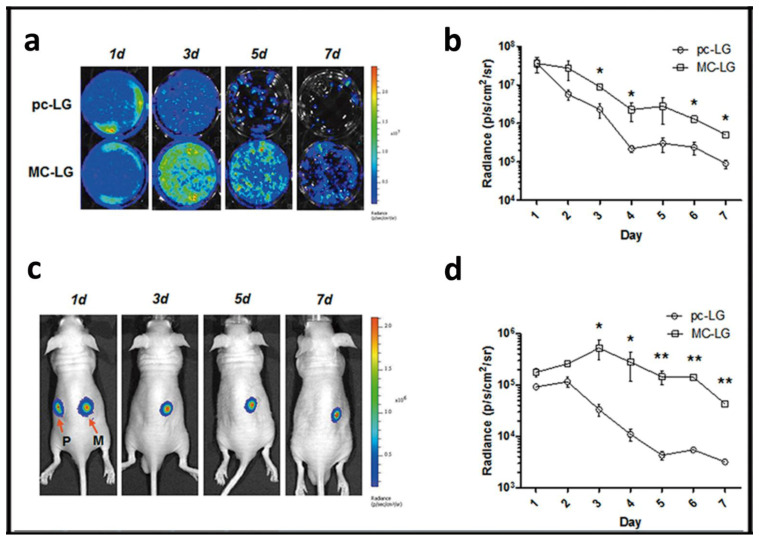
Minicircle approach to transfect mesenchymal stem cells (MSCs). (**a**) Bioluminescence images of MSCs transfected with pcDNA3.1-fLuc-2A-EGFP (pc-LG) or McCMV-fLuc- 2A-EGFP (MC-LG). (**b**) Quantification of bioluminescence signal emitted by the MSCs transfected with pc-LG (circle) or MC-LG (square). (**c**) Bioluminescence images of nude mice subcutaneously injected with MSCs transfected with pc-LG (P) and MC-LG (M) into left and right back. (**d**) Quantification of bioluminescence emitted from the injected area of mice. Reproduced with permission of [60].

Most of the strategies that have been developed by the research community to enhance the lasting effect of transgene expression are mainly focused on modifications of the genetic material to be delivered rather than on modification on the components of the non-viral vector formulation. For example, in the case of the *gene addition* approach, the previously described mcDNA technology not only reduces the cytotoxic effect but also represents a promising approach to prolong the therapeutic effect when this genetic cargo is combined with non-viral vectors [8]. The lack of bacterial backbone sequences, along with the low content of unmethylated CpG dinucleotides, reduces the activation of nuclear transgene silencing mechanisms, which finally results in a sustained transgene expression effect (Figure 6, [60]).

In addition, to prolong the effect, the transgenes of interest can be incorporated into the host genome by means of viral integrase or site-specific recombinase enzymes, or by the addition of transposable elements, such as transposons or “jumping” genes [55]. In any case, the translation into the clinic of these promising approaches to enhance the transgene expression effect is clearly conditioned by relevant safety issues such as the possible induction of insertional mutagenesis in the host cells with permanent consequences. Another approach that can be used to prolong the transgene expression effect consists of the addition of viral DNA sequences that allow plasmid replication outside the chromosomes. However, again, safety concerns can arise due to the viral DNA nature that is associated with immune responses and the risk of oncogenesis. As a safer alternative to the aforementioned viral sequences, mammalian scaffold/matrix attachment regions (S/MARs) can also be incorporated into pDNA to enhance the episomal replication of plasmids [61]. Episome sequences autonomously replicate plasmids that do not need to be integrated into the host genome to express the transgene, which minimizes the mutagenesis risk [62]. Episomal replicating plasmids are especially interesting to face tumor cells by gene therapy, where a vertical transfer of the therapeutic plasmids is particularly relevant in fast-dividing malignant cells [63]. Gene expression can also be prolonged by specific inhibition of gene silencing mechanisms of cells that hampers transgene expression [64]. Interestingly, artificial transcriptional activators can also be incorporated into therapeutic plasmids with appropriate promoters to modulate gene expression. In the case of the *gene inhibition* approach used to silence the expression of an altered gene with inhibitory sequences such as miRNA, siRNA or AON delivered by non-viral vectors, the effect is also transient, which requires repeated administrations [32]. In this case, a permanent correction of the altered gene can be achieved with the use of CRISPR/Cas9 technology. Such genome editing tools can be delivered by non-viral vectors complexed to different genetic constructs such as pDNA, mRNA or RNP complexes [19]. In any case, when time is a crucial factor, differences in anatomy, physiology, development and biological phenomena between animal labs and human beings should be also considered to extrapolate experimental results. For instance, it has been estimated that one lived day for rats is comparable to 30 lived days for humans [65].

## 4. Administration Route

**Figure 7:**
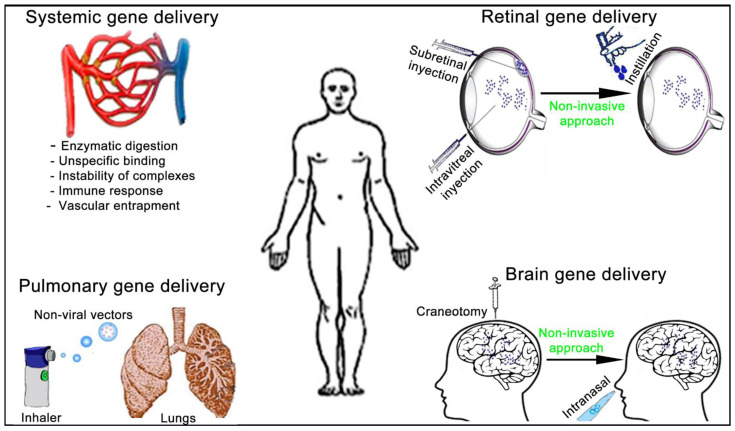
Schematic representation of the main administration routes to face systemic and tissue-specific human diseases by non-viral vectors gene therapy approach.

The administration route of non-viral vectors affects not only the previously described gene delivery efficiency and duration of gene expression but also the design of the formulations. To be active at the place of action, non-viral vectors need to overcome different biological barriers that strongly depend on both the administration route and the target organ (Figure 7).

The main and most studied administration route of non-viral vectors to face disseminated cancer or infectious diseases is the intravenous route [66]. The idea is to transport the genetic material to as many cancerous and infected cells as possible. However, efficiency of this interesting and ambitious approach is strongly limited by the enzymatic degradation that a genetic material can suffer from the point of entry [23]. In addition, positively charged non-viral vector complexes can interact by non-specific electrostatic attractions with negatively charged biological compounds such as serum proteins and blood cells, which limit their final performance [67]. Another concern to be aware of is the possible instability of such complexes in the extracellular biological medium at physiological conditions, where pH value, temperature and ionic stench, among many other factors, can result in the formation of aggregates along the exposure time [18]. Furthermore, the systemic administration at high volumes of non-viral vector complexes can trigger the host immune responses against some of their components, resulting in an inflammatory response, which can be more pronounced if administrations are repeated [68]. Finally, it is worth mentioning that endothelial cells of the vascular system constitute a relevant and effective biological barrier. This barrier can limit the size and number of non-viral vector complexes that can pass through it, therefore, reducing the transfection efficiency in the targeted tissues after a systemic administration [69]. 

To overcome the previously described systemic barriers, non-viral vectors can be structurally modified. For instance, the addition of positively charged protamine into non-viral vectors protects the genetic material from enzymatic digestion and, therefore, increases the transfection efficiency. This approach has also been successfully used with other lipid formulations such as solid lipid nanoparticles [70] or liposomes [71]. Other commonly used strategy to increase the stability of non-viral vectors complexes in biological fluids and reduce the immune response consists in the addition of poly(ethylene glycol) (PEG) chains or other hydrophilic polymers, such as poly(N-vinyl-2-pyrrolidone) (PVP), into the outer surface of complexes [72]. The neutral PEG chains produce a steric barrier against enzymatic degradation and avoid aggregation of non-viral vector complexes in systemic circulation [73]. In addition, PEG chains reduce the quantity of cationic lipids required to deliver a genetic material, which enhances biocompatibility of lipid formulations [74]. 

Another main route of administration to face devastating diseases that affect the lungs, such as cancer, cystic fibrosis or asthma, is the pulmonary route. In this case, intratracheal intubation can be used for gene delivery of pulmonary disease-oriented treatments. However, the non-invasive nature of the inhalation approach is preferable for clinical applications. Another advantage of this administration route includes the use of small doses, which in turn, reduces side effects by increasing drug concentration at the area of interest. In addition, inhalation can also be used for gene delivery to treat systemic diseases due to the quick absorption on the alveolar region of lungs [75]. In any case, the effectiveness of the inhalation process depends mainly on the amount of the genetic material that will finally reach the targeted region and the deposition pattern, which is deeply conditioned by the composition of the material to be delivered and by the device used for inhalation [76]. In this sense, relevant issues such as the characteristics of the drug delivered, the most adequate pattern for its delivery, the design of the device and its effectiveness need to be considered in detail to enhance the effectiveness of this appealing administration route. In the specific case of genetic material-based formulations, the main difficulty to reach the nucleus of target cells is related to the susceptibility of such molecules to be degraded by the hydrodynamic shear forces generated during aerosolization process, which result in a clear decrease of the efficiency compared to in vitro conditions [77]. Some interesting approaches to protect DNA during the aerosolization process consist of the incorporation of compounds such as bovine serum albumin (BSA), which stabilizes the supercoiled DNA [78], or the design of efficient aerosol delivery systems for genetic material, such as nebulizers, dry powder inhalers (DPIs), pressurized metered dose inhalers (pMDIs) or mechanical metered dose inhalers (mMDIs) [79]. 

Another relevant issue that hampers the delivery of genetic material to the lung of affected patients is the presence of specific biological extracellular barriers at this level such as the alveolar macrophages, the mucus and alveolar fluids, which make the diffusion of genetic material difficult due to mechanical obstructions and non-specific interactions [80]. In this case, excipients such as mucolytic agents, which improve the mobility of genetic material, or cell penetrating peptides (CPP), which enhance the cell internalization process, can be incorporated into non-viral vector formulations to enhance gene delivery efficiency into the lung [81].

However, there are other relevant organs such as the brain and the eye that, due to the essential functions that they perform, have developed additional extracellular barriers over many years of evolution to protect them against foreign agents such as the blood brain barrier (BBB) and the blood retinal barrier (BRB), respectively, in each organ [82,83]. Consequently, effective gene therapy approaches to treat both inherited and acquired diseases of the brain and the eye rely, currently, on the in situ administration of genetic material by invasive routes or in the systemic administration of non-viral vectors decorated with appropriate ligands to overcome such additional extracellular barriers [23]. In the case of the brain, most commonly used strategies to cross the BBB include the incorporation of receptor-mediated uptake of ligands such as insulin, transferrin or lactoferrin, since endothelial cells of the BBB express high quantity of receptors for such molecules [84]. Other interesting approaches to cross BBB consist of the addition of specific monoclonal antibodies that are recognized by receptors of the BBB into the non-viral vector formulations, or the transient mechanical disruption of the BBB permeability [84]. In the case of the eye, some studies have demonstrated that submicron-sized formulations based on liposomes or chitosans, with appropriate ligands such as annexin A5 or transferrin, can target the retina (which can be damaged in many inherited disease that affect eyesight), after non-invasive topical application into the conjunctival/scleral tissues [85,86]. Interestingly, non-invasive approaches have also been described to circumvent BBB. In this case, non-viral vectors based on 10 kDa polyethylene glycol (PEG)-substituted lysine 30-mers (CK30PEG10k) were able to deliver EGFP plasmid into the brain after intranasal instillation [87]. All these findings raise reasonable hope to treat human diseases that affect the eye and the brain by a safe and effective gene therapy approach based on non-viral vectors administered by non-invasive routes in the future.

## 5. Design of Experimental Conditions

To become clinically relevant, non-viral vectors must first meet some critical physicochemical and biophysical parameters that affect the transfection efficiency, such as size, morphology, superficial charge, thermal stability and rheological properties, to name just the most relevant ones [88,89]. Normally, this is the first set of experiments that are performed when designing non-viral vector formulations for gene delivery purposes. Even in this preliminary step, the design of experimental conditions to be evaluated needs to be considered in detail [48]. For instance, preferably, the nanometric particle size of non-viral vectors should be reported as hydrodynamic diameter by cumulative analysis [90] rather than by the area of the predominant peak measured by dynamic light scattering (DLS), normally in a Zetasizer instrument. However, to use this approximation correctly, and for comparative purposes, particle size distribution must follow a monomodal distribution, with a polydispersity index below 0.3. In addition, to be more sensitive to small numbers of aggregates or dust, such a particle size must preferably be reported as an intensity correlated function of the scattered light rather than by volume or number of particles distribution [91]. The particle size of formulations, along with the presence of possible aggregates, can also be analyzed with appropriate staining reagents such as uranyl acetate by different microscopic techniques, including transmission electron microscopy (TEM), cryo-TEM, scan electronic microscopy (SEM) or atomic force microscopy (AFM) [92]. However, with these approximations, the number of particles analyzed is more limited and not always provides a pretty correlation with the DLS technique, mainly due to the different sample manipulation processes between both approaches. Therefore, such microscopy-based approaches are normally used to provide evidence of the nanoparticles and evaluate their morphology, since this is another relevant parameter that affects the gene delivery process [93]. 

In addition to particle size, the superficial charge of non-viral vectors is another commonly studied physicochemical parameter that affects the electrostatic interactions with the genetic material [45]. Superficial charge value can also affect the interaction of complexes with cell membranes and the stability of colloidal dispersions. In this sense, high zeta potential values (either positive or negative) prevent aggregation among particles by electrostatic repulsions [90]. Such a parameter is normally reported as a function of the zeta potential value, which can also be measured in a Zetasizer instrument by lasser doppler velocimetry (LDV). In this case, the approximation model used to calculate zeta potential value is another relevant parameter to be considered, with the Smoluchowski equation being the most commonly one used [94]. 

Other parameters that directly affect both particle size and zeta potential values of non-viral vectors and make the profitably replication of these studies difficult for the sake of comparison are the pH value and the ionic strength of the measurement medium [95]. Moreover, the electrostatic interaction between non-viral vectors and the genetic material to obtain the corresponding complexes can be measured at a molecular level through the heat released when such binding occurs by isothermal titration calorimetry [96] or by agarose gel electrophoresis assays [48]. When this last technique is used, the protection capacity of genetic material by non-viral vectors from enzymatic degradation can also be evaluated. In any case, again, standardized protocols should be implemented to make such evaluations as unbiased as possible, since many parameters, such as the exposition time to the enzyme, the temperature, the composition of the buffer or the potential applied to run the electrophoresis assay, can impact the final results obtained. 

Once non-viral vectors show appropriate physicochemical parameters for gene delivery purposes, normally, and before performing any in vivo biological study, the gene delivery capacity is preliminarily evaluated in in vitro conditions. However, such in vitro studies that usually also consider the biocompatibility of the formulations, along with the cellular uptake and posterior intracellular trafficking analysis, are typically validated in experimental conditions that not always represent the in vivo environment [97]. For instance, in vitro results are normally obtained in homogenous cell populations that are exposed to non-viral vectors for long incubation times. Therefore, positive outcomes obtained in such simplified tests do not always guarantee success in further animal model validation assays [37]. A more realistic scenario, which better resembles an in vivo environment, is obtained when immortalized cell lines are substituted by primary culture cells [98]. In this case, due to the intrinsic characteristics of primary culture cells, transfection efficiency values normally decrease when compared to immortalized cell lines. Consequently, studies performed in primarily culture cells or in other difficult-to-transfect cell lines such as neurons are normally used to report the kind of cells that have been transfected by immunohistochemistry techniques rather than the transfection efficiency in quantitative terms [23,44]. 

**Figure 8:**
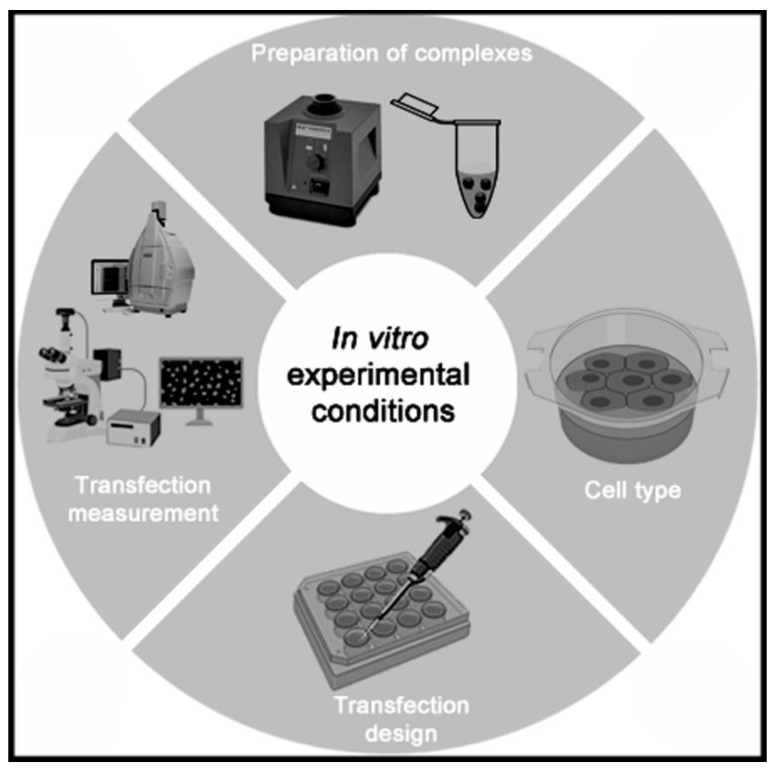
Brief schematic representation of relevant parameters that affect the in vitro performance efficiency of non-viral vectors.

Interestingly, more sophisticated in vitro scenarios, that better predict the in vivo performance of non-viral vectors, can be obtained using, for instance, microfluidic technology that resembles extracellular barriers of immune-privileged organs such as the eye or brain [99] or by application of the recent game changer 3D-bioprinting technology [100]. In any case, and as occurred with the physicochemical studies, the experimental conditions and protocols of biological assays performed in in vitro conditions should also be standardized for comparative purposes, since there are many variables that can influence the final results obtained [97] (Figure 8). 

For instance, the preparation of complexes plays a pivotal role in the final biological performance and depends on many factors such as the volumes or quantities used, the temperature, the order of components addition or the mixing technique, to name just the most relevant ones. In the case of non-viral vectors based on polymers, a stock aqueous solution is added to an appropriate volume of a solution, which contains the genetic material under vigorous vortex mixing for a short period of time at room temperature. However, normally, in the case of lipid formulations, the electrostatic interaction between the cationic lipid and the genetic material occurs under gentle mixture with an appropriate pipette to avoid destabilization of the lipid formulation [33]. In some cases, especially when large volumes of aqueous solutions are required to obtain the complexes, a hypertonic medium, based on the incorporation of appropriate isotonic agents such as mannitol, is added to get a final isotonic medium that will avoid the lysis of cells [37]. 

Moreover, transfection efficiency is a highly cell-dependent process, which is conditioned by the particular cellular uptake mechanism and the posterior intracellular trafficking pathway used before reaching the nucleus [45]. In this sense, the low division rate of quiescent cells, such as neurons or primary cells, hampers the entry of exogenous genetic material into the nucleus, challenging the transfection. On the contrary, phagocytic cells can be transfected more easily due to their native biological performance. Therefore, the concept of a unique universal non-viral vector optimized for all clinical applications is currently abandoned. In fact, the most accepted concept relies on the idea that the success of non-viral vectors into clinical practice depends on the development of gene delivery platforms based on multifunctional vectors specifically designed and tailored for each particular purpose [21]. In any case, and as a preliminary proof of concept, the first approach to evaluate transfection efficiency of non-viral vectors can be performed, for instance, in human embryonic kidney (HEK-293) cells, which is a common cell model easy to be transfected due to the specific biological characteristics of these particular cell line [37]. 

In close relation to the cell type, the transfection experiment conditions can also influence the results obtained. In this sense, some relevant parameters also need to be considered. For instance, the confluence density of the cells, the presence or absence of antibiotics and serum in the culture and transfection medium, or the volume and the quantity of genetic material that is going to be evaluated, which also depend on the area of the cell culture wells, should be standardized for comparative purposes. Finally, and as previously mentioned, transfection efficiency can be measured by different approaches, although normally reporter genetic material is used as a proof of concept before moving to therapeutic genetic material. In any case, slight overall readjustments need to be done in the non-viral vector/genetic material-used ratios due to the impact that both size and composition of the genetic material have on the transfection efficiency process [88]. 

Once positive outcomes have been obtained in in vitro test models, the next step is the evaluation of the performance into first small and later big animal models. Moreover, previously, ethical issues related to the use of animals at preclinical level need to be addressed. Once again, during this step, the complexes administered into in vivo studies might suffer slight modifications related to the volumes, doses or composition of the dilution medium, that depend overall on the administration route to be used [98]. In any case, success in animal models does not necessary guarantee clinical success into humans, since animal testing methods cannot fully address translation to human physiology [97]. In such clinical trials, the delicate balance between the potential benefits of the treatment and the possible associated risks is carefully evaluated under the supervision of a Gene Therapy Advisory Committee that will also deal with ethical issues [101].

## 6. Commercialization Process

**Figure 9:**
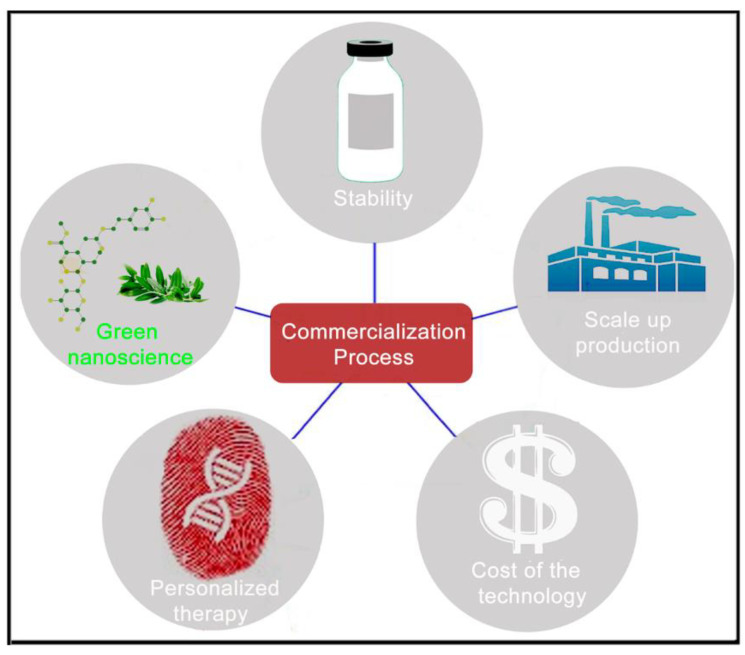
Schematic representation of relevant issues to consider across the commercialization way of non-viral vectors in gene therapy medical field.

Although from a scientific point of view, it is possible to deliver genetic material to specific cells by means of non-viral vector formulations, the regular application into medical practice of this gene therapy approach is highly conditioned by other factors that affect the commercialization process (Figure 9). 

For instance, the electrostatic interaction between non-viral vector formulations and genetic material cargo typically occurs in aqueous medium. However, despite the simplicity of this process, batch-to-batch variability can occur due to slight variations during the mixture procedure, which is not acceptable in a clinical setting [97]. In addition, systems based on aqueous suspensions are highly unstable to allow regular shipping and storage for long periods. In this sense, the recent RNA-based lipid vaccines, developed by both BioNTech and Moderna to face SARS-CoV-2 virus that causes COVID-19 infection represent a clear example of this issue. Interestingly, the development of dry powder formulations, where aqueous medium is removed, represents a promising approach to circumvent such instability issues. In fact, currently, many companies are working on the design and development of stable dry powder RNA-based lipid vaccines against SARS-CoV-2 virus. Such solid forms can be obtained by freeze drying (lyophilization), spray drying or spray congealing techniques [102]. In any case, this technological approach is not an easy matter, since both the components of the non-viral vector formulation and the genetic material can be degraded during the process. In the case of the lyophilization technology, appropriate cryoprotectant agents need to be incorporated into the non-viral vector formulation to avoid the formation of ice crystals during the freezing process. In addition, relevant parameters of the own technology, such as temperature, vacuum pressure and primary and secondary dry periods, need to be optimized to get a homogenous dry powder without residual moisture. The spray dryer technology can be used as an additional or complementary technique to lyophilization to also obtain a product in the form of a dry powder. In this case, the suspensions containing the complexes are absorbed and atomized, forming small vesicles on a stream of hot air, which causes the rapid solidification of these vesicles and their subsequent separation. As in the case of lyophilization, different amounts of excipients, such as mannitol, lactose or trehalose, can be added to improve the flow properties, avoiding collapse and agglomeration of the vesicles during the process, as well as the reconstruction of the vesicles during their rehydration. Likewise, different process parameters require to be adjusted, such as the flow rate of the air stream, the gas temperature or the atomization rate of the sample [103]. In the case of the spray congealing approach, a colloidal dispersion of complexes with appropriate excipients is atomized into liquid nitrogen with a nozzle [104]. Both the flow rate and the atomizing pressure need to be optimized to generate frozen droplets. Such frozen droplets are quickly transferred into a freeze dryer precooled at a shelf temperature. After the evaporation of the nitrogen, freeze drying is conducted at a vacuum level of 5 Pa to obtain a homogeneous, porous powder, without residual moisture that will be stored in glass vials for subsequent pulmonary administration by inhalation.

Another relevant step that hampers the commercialization process of gene delivery of advanced therapy medical products based on non-viral vectors is the scale-up production of these drugs, ideally under the Good Manufacturing Practices (GMP) guidelines to avoid any possible modification in their biological performance [105]. In this sense, it is worth mentioning the role that the regulatory agencies such as the European Medicines Agency (EMA) and the Food and Drug Administration (FDA) play to track the manufacturing process. These agencies specify the requirements needed to get the desired bioequivalence of formulations and guarantee the safety and efficacy of the new drugs [106]. Normally, such products are designed and elaborated by small and medium-sized enterprises that collaborate with academic groups. Although these consortiums are highly engaged in preclinical activities, they have limited manufacturing experience at industrial levels [105]. For instance, as commented before, non-viral gene therapy products are normally elaborated in research laboratories when genetic material is mixed with a lipid or polymer aqueous solution, obtaining small volumes of complexes, around 1 mL or even less [107]. In this scenario, the standardization of mixing parameters under GMP and the progress towards clinical application is practically unattainable [108]. In this sense, pilot plants represent an appealing approach to produce small volumes of this technology-based products, to gain knowledge about this technology, and later to design full-scale production systems that can lead in commercial products. 

In terms of treatment cost, and contrary to most of the conventional treatment drugs, gene therapy products focus their interest mainly on rare and specific disorders for a small population of patients, which raise relevant ethical questions [109]. In fact, the cost of many gene therapies approved to date is inversely related to the number of patients who could benefit from them, and in some cases, previously approved drugs for commercialization, such as Glybera, have been withdrawn from the market due to the low demand of patients and high cost of treatments [110]. At present, some of the most expensive marketed drugs, such as Luxturna or Zolgensma, are gene therapy products that use viral vectors to deliver the genetic material to the place of interest. In this sense, the costs to develop non-viral gene delivery vectors is clearly marginal if compared with viral counterparts, since mass production of viral vectors requires the development of expensive scalable and robust processes that affect, for instance, the studies on cell cultures or the amplification and purification steps. [111]. However, although being more affordable from an economical point of view, to reach clinical practice, non-viral vectors require multiple rounds of engineering and many chemical modifications, including the addition of stabilizing components or bioactive targeting ligands that increase their complexity and, therefore, the price of the formulations [105].

In any case, it is worth mentioning that the old idea of a unique conventional treatment for all patients affected by the same pathology is currently out of date. In fact, next-generation sequencing techniques (NGS) have completely revolutionized the genetic diagnosis of rare diseases, which enables the development of increasingly personalized therapies based on the genetic code characteristics of each individual [112]. This methodology allows a fast and accurate analysis of the altered genes at an affordable price. Such genetic information ensures clinical diagnosis, increases prognosis reliability, enables genetic counseling and opens the door to precision treatments. This is the idea of the new medicine that allows the design of personalized therapeutic treatments to correct the initial genetic defect in each patient and restore affected cell function [113]. A clear example of such personalized medicine is the recently developed Milasen product, a gene therapy-based drug created for a single six-year-old patient diagnosed with Batten’s disease, an inherited neurodegenerative disorder that leads to retinopathy, seizures and impaired mental and motor skills [114]. In this case, the genome of the patient was sequenced to identify the specific cause of this disease. Researchers found that neither of the two copies of patient’s major facilitator superfamily domain containing 8 (MFSD8) gene was functional. In one of them a pathogenic mutation was found and, in the other one, an insertion of a mobile genetic element was found, which affected the processing of the mRNA. Without a functional MFSD8 gene, the protein necessary for lysosomes to carry out recycling or processing activity of molecules in the cell cannot be produced. As a result, proteins or metabolic substances are accumulated progressively in cells compromising their functionality [114]. From the patient’s genetic information, researchers designed and administered specific antisense oligonucleotides to face the patient’s disease in a customizable approach. In less than a year and a half, researchers selected the most effective oligonucleotides, carried out all the tests on cells obtained from the patient and, after ex vivo evaluation of both efficiency and toxicity, obtained the pertinent institutional authorization to administer the drug. Obviously, the widespread extension of this approach to other human diseases by gene therapy products, based on non-viral vectors, could have a significant impact on the health parameters of the population and, as a consequence, decrease the economic burden that represents for the whole of society. 

**Figure 10:**
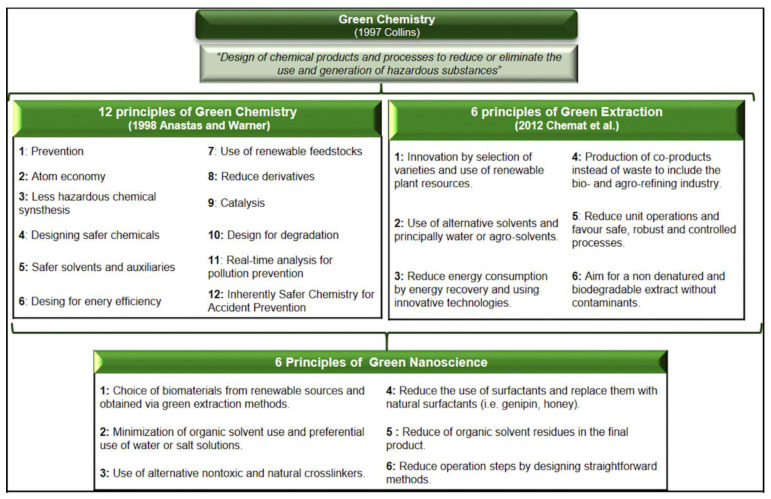
Definitions and principles of Green Chemistry, Green Extraction and Green Nanoscience. Reproduced with permission of [115].

Finally, an ideal scenario to get this appealing approach would also contemplate the elaboration of personalized non-viral vectors for gene therapy under the umbrella of the Green Chemistry concept (Figure 10).

Recent advances in the field of nanotechnology have made a great revolution in different biotechnological sectors, including medicine. Consequently, the intake of pharmaceutical products has increased considerably among the population over the years. However, the clinical and industrial application of this progress has been in the spotlight not only due to the safety risk and possible side effects associated with the use of these advanced drugs but also due to the negative ecological impact that their waste products can have on different aspects of the environment, such as water, soil and air. For instance, the high energy consumption during drug elaboration, along with the use of high amounts of hazardous organic solvents, has a relevant impact in such issues. In addition, most of the drugs and metabolites in wastewater reach the wastewater treatment plants (WWTPs), which are not specifically designed to eliminate these types of compounds. This becomes the main source of entry into the natural environment, which is of utmost relevance due to the possible toxicological risks the drugs can produce [116]. Consequently, the field of Green Nanoscience/Nanotechnology has emerged as a revolutionary strategy to prevent any associated toxic and negative effect on the environment, through the implementation of sustainable and ecologically friendly processes across the whole lifecycle from the extraction of nanomaterials and active compounds (Green Extraction) to the application of the final nanoformulation [115,117]. Some of the Green Nanoscience/Nanotechnology principles include the election of biomaterials from renewable sources obtained by Green Extraction methods, the replacement of organic solvents by water or salt solutions, the use of alternative nontoxic and natural crosslinker, the preferential use of natural tensioactives or the reduction of operating steps designing straightforward smethods [118,119,120]. In any case, notwithstanding the success of Green concepts across inorganic nanoparticles, their application into organic nanoparticles to design and elaborate non-viral vectors for gene therapy is still far away to be a regular practice in the research community.

## 7. Conclusions

The substantial progress that has been achieved during the past years in different research areas associated with the design of novel gene delivery systems, along with the gain of knowledge acquired in genomics and structural biology, has raised reasonable hope to consider the regular application into medical practice of non-viral vectors as gene delivery systems to face many devastating diseases. Compared to viral vector counterparts, non-viral vectors show relevant advantages. For instance, they are less limited by the genetic packing capacity, are better tolerated by the host immune system and their production is easier and cheaper because they do not have the limitations associated with biological agents. In fact, non-viral vectors are classified as drugs rather than as biological agents by the regulatory authorities, which enhances the clinical translation. However, the reality is that non-viral vectors have been poorly translated into clinical practice. Many concerns that hamper clinical practice of non-viral vectors include low transfection efficiency, short gene expression effect, difficulties to reach the target cells after in vivo administration, lack of implementation of standardized protocols for an unbiased evaluation of the performance of non-viral vectors and issues associated to the commercialization process. Although there is no universal non-viral vector for all purposes, in our opinion, lipid non-viral vectors are the closest to reach clinical translation in the gene therapy field. A cutting-edge example is represented by current lipid nanocarriers that have been successfully developed to face the COVID-19 disease by an oligo-based therapeutic approach delivering synthetic mRNA as a nanovaccine. This strategy raises hope to use a gene therapy approach to face diseases with lipid nanoparticles delivering plasmid DNA in the near future. In any case, the success of this appealing approach clearly depends on the development of other game-changing technologies such as big data, tissue engineering or 3D bioprinting to face genetic diseases from a safe, affordable and multidisciplinary point of view.

## Figures and Tables

**Table 1:** Scheme of essential characteristics and properties of main polynucleotides used in oligo-based gene therapeutics and gene therapy.

Mechanism of Action	Polynucleotide	Chemical Structure	Lasting Effect	Place of Action
Gene addition	Plasmid (dsDNA)	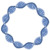	Transient	Nucleus
Gene addition	mRNA (ssRNA)	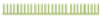	Transient	Cytoplasm
Gene inhibition	miRNA (ssRNA)	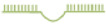	Transient	Cytoplasm
Gene inhibition	siRNA (dsRNA)	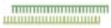	Transient	Cytoplasm
Gene inhibition	AON (ssDNA/RNA)	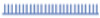	Transient	Cytoplasm/Nucleus
Genome editing	CRISPR/Cas9 Plasmid (dsDNA)	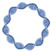	Permanent	Nucleus
Genome editing	CRISPR/Cas9 mRNA (ssRNA)	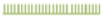 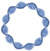	Permanent	Cytoplasm/Nucleus
Genome editing	CRISPR/Cas9 Ribonucleoprotein	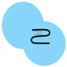	Permanent	Nucleus
